# A modified anchored suturing technique for enhanced flap stability in periodontal plastic surgery: A case series

**DOI:** 10.34172/japid.025.3954

**Published:** 2025-10-14

**Authors:** Moein Khojaste, Farid Shiezadeh, Zahra Moslehitabar, Masoud Amiri Moghaddam

**Affiliations:** ^1^Department of Periodontics, School of Dentistry, Mashhad University of Medical Sciences, Mashhad, Iran; ^2^Dental Research Center, Mashhad University of Medical Sciences, Mashhad, Iran

**Keywords:** Connective tissue, Gingival recession, Plastic surgery, Suture anchors, Suture technique

## Abstract

**Background.:**

Gingival recession is a common mucogingival condition that may cause esthetic concerns, root sensitivity, and functional problems. Tunneling techniques with connective tissue grafts (CTGs) are well established for root coverage and esthetic preservation. Various suspensory sutures have been proposed to stabilize coronally advanced flaps. The butterfly suture is a modified anchored approach intended to provide simultaneous stabilization of interproximal and midfacial areas. This case series describes the clinical application and short-term outcomes of this technique.

**Methods.:**

Three systemically healthy patients (two males and one female, aged 20–45 years) with Cairo RT1 and RT2 recession defects were treated using a tunneling technique combined with CTG and stabilized with the butterfly suture. The patients were followed for 6 weeks, and outcomes were assessed descriptively.

**Results.:**

Nine teeth were treated in the three patients. Seven defects achieved complete root coverage (CRC), and two achieved partial root coverage (PRC). Healing was uneventful in all cases, with no complications such as infection or necrosis. The patients reported satisfaction with the esthetic outcomes and resolution of dentin hypersensitivity.

**Conclusion.:**

Within the limitations of this small case series, the butterfly suture provided stable coronal advancement and favorable root coverage outcomes. This technique may represent a simple and efficient alternative in tunneling procedures. Larger controlled studies with longer follow-up and patient-reported outcomes are necessary to validate its effectiveness.

## Graphical Abstract

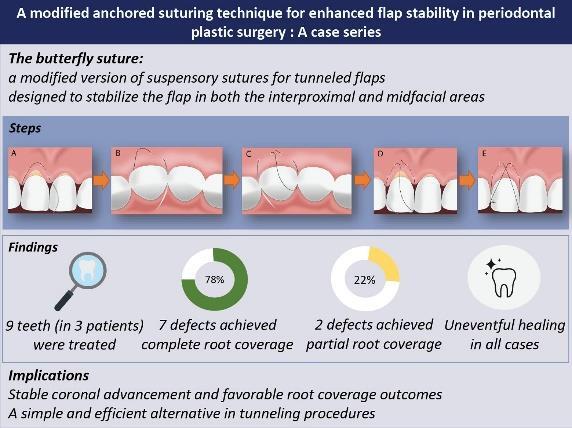

## Introduction

 Root coverage procedures are undertaken to improve esthetics and reduce dental sensitivity. A variety of treatment approaches with varying levels of success are available. Previous studies have recorded the treatment of gingival recessions using coronally advanced flap, envelope, pouch, and tunnel techniques, often including connective tissue grafts (CTG).^1-3^ Among these different methods, tunneling techniques that preserve papillary integrity have been supported to improve blood supply, facilitate healing, and enhance esthetic results.^4,5^ Compared to coronally advanced flap in the tunneling techniques, it is more challenging to stabilize the flap and soft tissue graft in a coronal position due to limited access. Therefore, several suspensory (sling) sutures have been recommended to secure the movable flap to the desired coronal position and use the immobile anchors to maintain its position during the healing period. Some suitable anchors in the oral cavity include tooth contacts, implants, composite resin, and orthodontic brackets.^6^

 Proximal tooth contacts splinted with composite resin material before surgery could be an option for anchoring sutures, such as the “vertical double-crossed suture” technique described by Zuhr et al^7^ This suture can maximize coronal displacement of the entire buccal soft tissue complex by interdental anchoring. Also, crossing the suture around the contact point provides additional compression to the underlying soft tissue graft, further improving graft nourishment during the early wound healing period.^6,7^

 Stabilizing the flap only in the interproximal area without considering the midfacial portion may cause some flap micromovements in the midfacial part and endanger the final root coverage success.^8,9^ Other suturing methods have been described to stabilize the flap in the midfacial area, rather than interproximal, including those discussed below:

###  “Coronally Anchored Suturing” Technique

 The “coronally anchored suture” presented by Zadeh^10^ includes a horizontal mattress suture 2‒3 mm apically to the free gingival margin within the keratinized gingiva, with the knot placed at the mid-coronal surface of the tooth and bonded with composite resin. This method will lead to coronal fixation of the mid-buccal portion. Still, some potential problems with this technique include patients reporting visible sutures in the middle of the tooth surface.^9^ Also, anchoring the flap on the buccal tooth surface may drag the flap in the buccal direction.^6^ Furthermore, placing suture thread horizontally under the keratinized gingiva may disrupt the optimal flap adaptation.^6^

###  “V-Reverse” Suturing Technique

 The “v-reverse” suture suggested by Chacón Ramírez et al^9^ improves flap stabilization in the mid-facial portion, which is the most critical area that should be secured to achieve complete root coverage (CRC).^9,11^ In this method, the needle penetrates the graft and buccal flap from internal to external surface 3 mm apical to the gingival margin within keratinized tissue in the midfacial area. This technique is not suitable for the thin gingival phenotype.

###  “Subpapillary Continuous Sling” Suturing Technique

 The “subpapillary continuous sling” suture described by Allen^12^ includes engaging the flap and graft 3 mm apical to the soft tissue margin in the mid-facial portion. Consequently, it maintains the graft and tunnel flap in the coronal position.

###  “Belt and Suspenders” Suturing Technique 

 The “belt and suspenders suture” technique presented by Ronco and Dard^13^ consists of a modified, anchored horizontal and vertical mattress sutures. It uses the proximal contact as an anchorage for the coronal displacement of both papillary and mid-facial parts of the mucogingival complex. This technique is suitable for wide and asymmetric recession defects. However, using a large number of sutures, including two modified vertical mattress sutures and one modified horizontal mattress suture, is generally regarded as undesirable because it can contribute to tissue trauma.^13^

 The butterfly suture technique is a modified version of suspensory sutures designed to stabilize the flap in both the interproximal and midfacial areas. In this technique, at the mid-facial surface of the flap, two overlapping suture threads are pulled diagonally due to their anchorage around the interdental contacts. These oblique threads exert coronal and horizontal force on the engagement point. Thus, the butterfly suture technique accommodates two coronal traction vectors at the subpapillary points and bilateral horizontal traction in the tissue apical to the tooth line angles, which could help re-create the natural scalloping of the gingival margin.^14,15^ Subsequently, this suturing technique reinforces coronal fixation of the mid-facial part of the flap. It enhances intimate contact between the possible graft, the gingival flap, and the hard buccal tooth surface in this area. Theoretically, the butterfly suture may reduce unfavorable micromotions in the buccal gingivopapillary complex and improve advancement efficacy.

## Methods

 This descriptive case series was conducted at the Department of Periodontics, Mashhad University of Medical Sciences, in accordance with the Declaration of Helsinki (2013 revision). The study protocol was approved by the institutional Ethics Committee (IR.MUMS.REC.1403.356). Three systemically healthy patients (two males and one female, aged 20, 40, and 45 years) presenting with Cairo RT1 or RT2 recession defects were included.^16^ Inclusion criteria were: age ≥ 18 years, good oral hygiene, and localized recession defects requiring root coverage. Exclusion criteria were: smoking, systemic contraindications to periodontal surgery, active periodontal disease, or pregnancy. Written informed consent was obtained from all the participants. Clinical parameters were recorded before surgery, including recession depth (RD), recession width (RW), probing depth (PD), keratinized tissue width (KTW), gingival phenotype (thin or thick), and cementoenamel junction (CEJ) condition (detectable or step).^17^ All measurements were performed using a UNC-15 periodontal probe. The primary outcome was root coverage categorized as CRC (complete) or PRC (partial). Healing characteristics and postoperative complications were documented descriptively.

###  Surgical Technique

 All the procedures were performed under local anesthesia (2% lidocaine with 1:80,000 epinephrine). Exposed root surfaces were thoroughly debrided and planed with hand instruments and fine finishing burs to obtain a clean, smooth substrate for graft adaptation.

*Interdental anchorage (composite splinting):* Before starting the surgery, the interdental contact points of the affected adjacent teeth were temporarily splinted using a flowable, light-curing composite resin material. Because of the natural undercuts in the interproximal regions, no additional etching or bonding was required in most cases. This step provided stable interdental anchorage for the sutures and helped maintain the flap in a coronal position during the healing period.

*Tunnel preparation and grafting:* A minimally invasive tunneling approach was performed through a limited vestibular access with intrasulcular extensions to mobilize the buccal flap and completely release the papillae,^18^ allowing passive coronal advancement of the flap–papilla complex. After tunnel preparation, a CTG was inserted beneath the tunnel flap and secured with lateral sutures.^19^ Fine, absorbable sutures were preferred for CTG fixation underneath the flap, as they minimize mucosal irritation and patient discomfort, especially in cases where the suture thread is exposed to the oral mucosa.^20^ In the cases presented in this study, we used 6-0 coated polyglycolate sutures for this purpose. For butterfly sutures, we used nylon threads due to their superior knot security and reduced plaque accumulation, although similar outcomes can be achieved with various monofilament suture materials.

###  Butterfly Suture (Step-by-Step)

**Figure 1:**
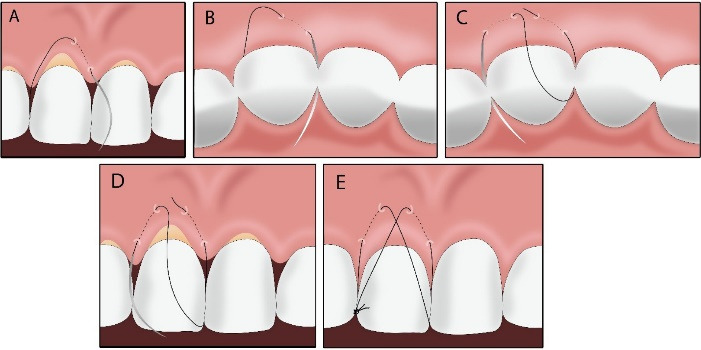
Schematic illustration of the step-by-step procedure of the butterfly suture. (A) The needle engages the buccal flap 3 mm apical to the gingival margin in line with the mesial line angle of the tooth and then reappears in line and apical to the mesial papilla tip. (B) The needle passes to the palatal side under the contact point and then returns to the buccal surface without pinching any tissue. (C) Again, the needle engages the buccal flap 3 mm apical to the gingival margin in line with the distal line angle and then reemerges apically to the tip of the distal papilla. (D) The needle passes under the distal contact point and wraps around it, and then returns to the buccal surface. (E) Then the knot is performed and tightened over the contact point.

**(A)** The needle was inserted through the buccal flap 3 mm apical to the gingival margin in line with the mesial line angle within keratinized tissue and directed coronally and mesially to emerge apical to the mesial papilla tip (Figure 1A).

**(B)** The suture was slid beneath the mesial contact point, wrapped around it, and returned to the buccal surface without engaging soft tissue (Figure 1B).

**(C–D)** The same sequence was repeated at the distal line angle: the needle was passed 3 mm apical to the margin, was guided coronally and distally to emerge apical to the distal papilla tip; then, it was passed under the distal contact, wrapped, and returned to the buccal surface (Figure 1C, 1D).

**(E)** A single knot was tied over the contact point until the intended coronal advancement was achieved; when necessary, the CTG beneath the tunnel was lightly engaged for additional stabilization (Figure 1E).

###  Postoperative Care 

 Sutures were removed after two weeks. The patients were instructed to avoid brushing the surgical sites for three weeks, use 0.12% chlorhexidine mouthrinse twice daily during this period, follow a soft diet, and take standard analgesics as needed.

## Results

###  Participants and Defects Treated

 Three patients (male, 20 years; male, 40 years; female, 45 years) presented with a total of nine recession defects in the mandibular anterior and posterior sextants. Table 1 presents defect characteristics and periodontal parameters. Follow-up evaluations were performed at six weeks for Case 1 and at four weeks for Cases 2 and 3.

###  Brief Case Summary

**Figure 2:**
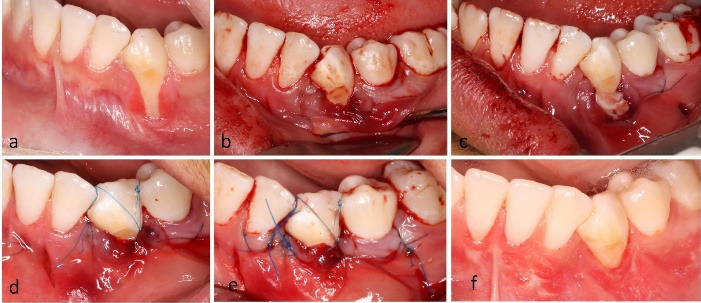
Clinical case 1: single tooth recession and thin periodontal phenotype. (a) Clinical presentation of Cairo RT2 gingival recession of the mandibular left canine. (b) Full-thickness tunnel preparation through 2 vertical vestibular incisions (modified VISTA approach). (c) CTG was inserted into the tunneled flap and fixed with simple sutures. (d) Butterfly suture was applied for the canine tooth. (e) Immediate postsurgical presentation. (f) Clinical presentation 6 weeks after surgery

*Case 1.* A 20-year-old male with a thin gingival phenotype presented with a Cairo RT2 recession at tooth #33. A modified VISTA tunnel was prepared, a subepithelial CTG was inserted, and flap stabilization was achieved using the butterfly suture with 6-0 nylon. At six weeks, CRC was observed with an apparent gain in gingival thickness (Figure 2).

**Figure 3:**
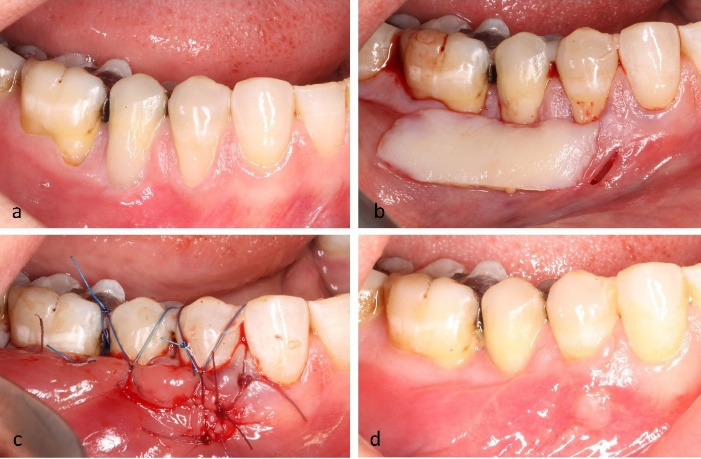
Clinical case 2: Multiple adjacent recessions with composite resin restorations. (a) Clinical presentation of Cairo RT1 defects on the mandibular right premolars. (b) CTG positioned in the correct surgical place. (c) Postsurgical presentation. (d) Clinical view 6 weeks after surgery

*Case 2.* A 45-year-old female presented with RT1 recessions at teeth #44 and #45 and cervical restorations, which were removed prior to surgery. The cementoenamel junctions were reconstructed, followed by tunnel preparation and insertion of a CTG. Both teeth were stabilized with 5-0 nylon butterfly sutures. At six weeks, CRC was achieved (Figure 3).

**Figure 4:**
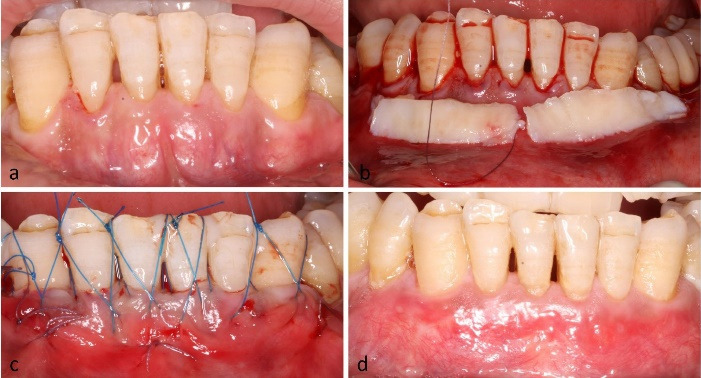
Clinical case 3: Multiple adjacent recessions. (a) Clinical view of multiple Cairo RT2 gingival recessions in the anterior sextant of the mandible. (b) CTGs placed in the correct surgical position. (c) Immediate postoperative view. The left lateral tooth does not have a butterfly suture, as the desired advancement was achieved with butterfly sutures in the central and canine regions. (d) Clinical presentation 6 weeks after surgery

*Case 3.* A 40-year-old male presented with multiple RT2 recessions in the mandibular anterior region. Three vertical vestibular accesses were made, and two CTGs were introduced beneath the tunnel. Butterfly sutures were applied at each tooth. At 4 weeks, CRC was obtained at the canines and lateral incisors, while partial coverage was noted at the central incisors (Figure 4).

###  Healing and Root Coverage Outcomes

 All the surgical sites healed uneventfully without necrosis, infection, or dehiscence. The patients reported only minor discomfort, which resolved within the first postoperative week, and none expressed aesthetic concerns about the visibility of sutures. Across the nine treated teeth, CRC (CRC) was observed in seven, while partial root coverage (PRC) occurred in two.

**Table 1:** Clinical evaluation of gingival recession and different postoperative root coverage outcome assessments

**Case number**	**Age**	**Sex**	**Tooth number**	**recession depth (mm)**	**recession width (mm)**	**PD (mm**)	**KTW (mm)**	**RT**	**Gingival** **phenotype**	**CEJ/step**	**Follow-up** **(weeks)**	**Root** **coverage**
1	20	Male	33	4	2	1	0	2	Thin	B/ +	6	CRC
2	45	Female	44	2.5	2	0.5	1	1	Thick	B/ +	4	CRC
			45	3.5	2	0.5	0.5	1	Thick	B/ +	4	CRC
3	40	Male	31	1	3	1	1.5	2	Thick	A/-	4	PRC
			32	1	2	1	1.5	2	Thick	A/-	4	CRC
			33	1	4	1	1	2	Thick	A/-	4	CRC
			41	2	3	1	1.5	2	Thick	A/-	4	PRC
			42	2	2	1	1.5	2	Thick	A/-	4	CRC
			43	1	3	1	1	2	Thick	A/-	4	CRC

PD: probing depth; KTW: keratinized tissue width; RT: recession type; CEJ: cementoenamel junction; PRC: partial root coverage.

## Discussion

 The suturing technique is important for optimal surgical outcomes in plastic periodontal surgeries and should provide two vital requisites: adequate wound stabilization and close contact with the affected tissues.^21^ Regarding coronal repositioning techniques, the suture should effectively secure the flap in a coronal position and maintain its stability throughout the entire initial healing period. To achieve these purposes, various suturing methods have been proposed, each targeting key improvements such as minimal invasiveness, shorter time, better handling properties (e.g., ease of knotting), cost-effectiveness, reduced technique sensitivity, and increased patient compliance.^22^ The butterfly suture technique can be suggested as an appropriate suturing method in a diverse range of clinical circumstances where the tunnel technique is indicated, including gingival recession coverage,^23^ phenotype modifications,^24^ and soft tissue ridge augmentations.^5,25^ This technique fulfills crucial requirements for the success of the mentioned surgical procedures. Proper coronal mobilization and fixation of the buccal soft tissue complex can be accomplished due to the coronal position of the anchoring area. The anatomic position of contact points, which are placed coronally and palatally/lingually to the surgical site, provides sufficient vertical traction to the buccal soft tissue complex and gentle compression of the tunneled flap to the underlying tissues.^13^ This technique applies force to two tissue points on each side, leading to a better and more effective distribution of forces within the tissue. Additionally, engaging the more medial portions of the tissue and applying forces diagonally facilitate better flap advancement. In comparison with the “vertical double-crossed suture”, which is a popular contact-based approach, the butterfly suture offers some advantages. It does not require lingual or palatal needle penetration, simplifying the procedure and reducing patient discomfort. Furthermore, the vertical double-crossed suture engages only the papillary region and does not apply force to the midfacial portion of the flap, which is often a challenging area to stabilize in tunnel procedures. In contrast, the butterfly suture engages both the papillary and midfacial portions and provides more effective coronal advancement of the midfacial aspect of the flap. Compared with the “belt and suspenders” technique,^13^ which requires three separate sutures and knots for the mesial, distal, and mid-facial regions, the butterfly suture requires only one suture and a single knot. This allows simultaneous stabilization of the mesial and distal papillae and the mid-facial region of the flap. The reduced number of knots shortens surgical time, decreases tissue trauma and surgical invasiveness, and minimizes the risk of plaque accumulation and postoperative irritation.^26^ The “coronally anchored suture” presented by Zadeh^10^ is an innovative approach for mid-facial flap stabilization. While the coronally anchored suture mainly targets coronal positioning of the mid-facial flap, the butterfly suture simultaneously addresses both the mid-facial and interdental papillae regions, potentially leading to a more balanced coronal repositioning of the entire flap. Unlike the coronally anchored suture, the butterfly suture does not require etching and bonding on the facial tooth surface. Additionally, since the coronally anchored suture relies on buccal tooth anchorage, the flap may be slightly pulled buccally,^6^ whereas the butterfly suture avoids this and helps maintain better flap adaptation.

 One disadvantage of this suturing technique is that, if the knot is placed above the proximal contact, excessive pressure during mastication can cause the knot to tear and the suture to open. If the patient’s occlusal forces are too heavy at the contact areas, placing the knot on the buccal aspect of the contact area is recommended. Also, applying a minimal amount of flowable composite resin on the suture thread can help prevent it from tearing during mastication. Aesthetic concerns arising from the appearance of suture threads on the buccal tooth surface are another limitation of this suturing technique. Although we did not receive any aesthetic complaints from the patients in this study, it should be considered a potential disadvantage, particularly in the anterior maxilla.

 Weighing its advantages and limitations, the butterfly suture technique offers a viable option for clinicians in root coverage procedures, facilitating simultaneous advancement of the interproximal and mid-buccal areas with a single knot in a straightforward suture pattern.

 One limitation of this study is that the clinical parameters were measured and presented descriptively, without statistical analysis. In addition, patient-reported outcomes, such as esthetic satisfaction and discomfort, were not assessed, which represents an important limitation. Future studies should incorporate these measures to better reflect the patient’s perspective and provide a more comprehensive evaluation of treatment outcomes.

## Conclusion

 The butterfly suture can be considered an appropriate suturing technique in various clinical scenarios where tunneling flap preparation is indicated. This method can meet a series of central demands, such as proper coronal displacement and stabilization of graft and flap. Future studies, including long-term follow-up of clinical cases, are required to validate this innovative approach and compare its results with previously described conventional suture methods used in a tunneling approach.

## Competing Interests

 The authors declare that they have no competing interests.

## Consent for Publication

 Written informed consent was obtained from all the patients for the publication of their clinical details and images. Patients were informed that anonymized data would be used for scientific reporting, and they agreed to the inclusion of their cases in this study.

## Data Availability

 The data of this study can be presented upon reasonable request from the corresponding author, Zahra Moslehitabar, at the Department of Periodontics, School of Dentistry, Mashhad University of Medical Sciences, Vakilabad Blvd., Mashhad, Iran.

## Ethical Approval

 This study was performed in accordance with the Helsinki Declaration of 1975, as revised in 2017, and was approved by the Mashhad University of Medical Sciences Ethics Committee for research under the code: ir.mums.rec.1403.356. All patients who participated in this study provided written informed consent
